# Optimization of the process parameters for reduction of gossypol levels in cottonseed meal by functional recombinant NADPH-cytochrome P450 reductase and cytochrome P450 CYP9A12 of *Helicoverpa armigera*

**DOI:** 10.1186/s13568-019-0823-4

**Published:** 2019-07-05

**Authors:** Cheng Chen, Yan Zhang, Wenhui Pi, Wenting Yang, Cunxi Nie, Jing Liang, Xi Ma, Wen-ju Zhang

**Affiliations:** 1 College of Animal Science and Technology, Shihezi University, Shihezi, 832000 Xinjiang China; 2 School of Chemistry and Chemical Engineering, Shihezi University, Shihezi, 832000 Xinjiang China; 3 State Key Laboratory for Sheep Genetic Improvement and Healthy Production, Xinjiang Academy of Agricultural and Reclamation Sciences, Shihezi, 832000 Xinjiang China; 4 State Key Laboratory of Animal Nutrition, College of Animal Science and Technology, China Agricultural University, Beijing, 100193 China

**Keywords:** *Helicoverpa armigera*, Cytochrome P450, CYP9A12, Gossypol, Detoxification

## Abstract

Gossypol is a toxic polyphenolic product that is derived from cotton plants. The toxicity of gossypol has limited the utilization of cottonseed meal (CSM) in the feed industry. The gene, *Helicoverpa armigera CYP9A12*, is a gossypol-inducible cytochrome P450 gene. The objective of our study was to obtain the functional recombinant *H. armigera* CYP9A12 enzyme in *Pichia pastoris* and to verify whether this candidate enzyme could decrease gossypol in vitro. Free and total gossypol contents were detected in the enzyme solution and in CSM. The *H. armigera* CYP9A12 enzyme degraded free concentration of gossypol. After optimization of the single-test and response surface method, free gossypol content could be decreased to 40.91 mg/kg in CSM by the *H. armigera* CYP9A12 enzyme when the initial temperature was 35 °C, the enzymatic hydrolysis time lasted 2.5 h, the enzyme addition was 2.5 mL, and the substrate moisture was 39%.

## Introduction

The presence of toxic free gossypol in cottonseed meal (CSM) greatly limited its efficient use in animal feed (Matlin and Zhou 1984; Matlin et al. 1988; Yildirimaksoy et al. 2004). The toxicity of gossypol results from two active aldehyde groups, which also have applications in pharmaceutical and therapeutic methods for such diseases as cancer (Dodou 2005). Our previous studies demonstrated that microbial detoxification of CSM can effectively eliminate its toxic effect (Zhang et al. 2006a, b), but the production efficiency was low for a long fermentation period. The hypothesis was that gossypol-degrading enzymes could play a key role during gossypol detoxification. However, studies on the gossypol-degrading enzyme and its functional gene are rare. Therefore, the target enzyme protein could not be obtained from a heterologous translational system based on the functional gene of that microbial enzyme.

The induction of the *H. armigera* P450 monooxygenase *CYP6AE14* and *CYP9A12* genes (Mao et al. 2007; Celorio-Mancera et al. 2011; Zhou et al. 2010) were possibly involved in the resistance and metabolism of gossypol (Jia et al. 2008; Kong et al. 2010; Krempl et al. 2016a, b). The expression of *H. armigera* CYP9A12 and CYP9A14 in yeast can detoxify xenobiotics (Yang et al. 2008), but metabolism of gossypol was not studied. The growth and development of *H. armigera* larvae was retarded after they were fed dsRNA *CYP6AE14* transgenic plants (Mao et al. 2007, 2011). The NADPH-cytochrome P450 reductase (CPR) was essential to help cytochrome P450 monooxygenase detoxify the substrates and xenobiotics (Guengerich et al. 2009) because the coexpression of house fly NADPH P450 reductase with *H. armigera* CYP6AE14 (Tao et al. 2012) and CYP6AE14 microsomes (Krempl et al. 2016a) resulted in epoxidation activity towards aldrin.

In our previous study, the functionally coexpressed *H. armigera* CYP9A12 and its CPR had been obtained in the *Pichia pastoris* system. The *H. armigera* CYP9A12 microsomal protein could significantly decrease gossypol concentration in enzyme solution and accelerate oxidization of the free gossypol intermediate metabolites G1 (m/z 265) and G2 (m/z 293) to the final product G0 (m/z 209) and G0′ (m/z 249) (Chen et al. 2019). In this study the effect of the *H. armigera* CYP9A12 microsomes on gossypol detoxification in vitro and in the CSM was validated. The optimization of the single-factor test and response surface methodology was used to determine the optimal conditions for gossypol-enzymatic detoxification in CSM to be utilized in the feed industry in the future.

## Materials and methods

### Construction and expression of the recombinant plasmids *H. armigera* CPR-2A-α-factor signal-CYP9A12

The *H. armigera CPR* (Accession Number: HM347785.1) and *CYP9A12* (Accession Number: AY371318.1) were amplified from *H. armigera* midgut cDNA (Chen et al. 2019) by specific primers and then cloned into the pGEM-T easy vector (Promega, Madison, Wisconsin, USA). The 2A sequence from the foot-and-mouth disease virus (FMDV) (Donnelly et al. 2001) was inserted between the *CPR* and the *α*-*factor secretion signal* sequences to obtain the functional protein independently. The *CPR*, the *2A*-*α*-*factor signal*, and *CYP9A12* fragments were combined with linearized *pPICZαA* vector using Gibson assembly which was described in our previous study in details (Chen et al. 2019).

The constructed plasmid were linearized and subsequently transformed into *P. pastoris* GS115 competent cells by electroporation. The transformants were selected by Zeocin antibiotic plate. The prepared buffered glycerol-complex medium (BMGY) and buffered methanol-complex medium (BMMY) were used to the yeast growth and induction. The microsomes were isolated by differential centrifugation of the cell homogenate. The separated *H. armigera* monooxygenase and reductase were detected by SDS-PAGE and the *H. armigera* CYP9A12 proteins were specifically detected with an anti-His-tagged mouse polyclonal antibody (CST, USA) and an eECL Western blotting kit (CW Biotech, Beijing, China) (Chen et al. 2019).

### Validation of the *H. armigera* CYP9A12 for the detoxification of gossypol from CSM

A total of 2.5 mL *H. armigera* CYP9A12 enzyme (equivalent to 575 U/mg enzyme activity unit) was added to 50 g CSM at an initial temperature of 30 °C, substrate moisture of 50% and was incubated for 4 h. The conditions of the *Candida tropicalis* group were fermentation time 48 h, fermentation temperature 30 °C, inoculum volume 5% (v/w), substrate moisture content 50%, natural pH, and three replicates per treatment (Zhang et al. 2006a, b). The control group (without any enzyme) was inoculated with the same volume of distilled water. After the reaction was finished, the samples were dried in a vacuum freezer and crushed through 60-mesh screens for detection (Firestone 2003).

CSM or treated CSM (500 mg) was accurately weighed and ultrasonically extracted with 70% acetone and butanone, respectively, at room temperature for 1 h. The total and free gossypol contents in the CSM were determined using HPLC as described above (Chen et al. 2018).

### Optimization of the *H. armigera* CYP9A12 enzymatic hydrolytic conditions for the CSM

#### Single-factor test

The detoxification conditions of the CSM enzymatic hydrolysis primarily include temperature, time, the amount of enzyme added, and substrate moisture. Use of the proper enzymatic hydrolysis conditions could not only improve the quality of CSM products and save resources but also increase production efficiency. The single-factor design was used to optimize the conditions of the *H. armigera* CYP9A12 enzyme in CSM enzymatic detoxification. The level conditions were based on the single factor test. CSM (50 g) was weighed into the corresponding number conical bottle. Sterilized water was added according to the ratio of the material to water (35%, 40%, 45% and 50%) (Table 1). The enzymatic hydrolytic temperature and time were set based on different levels in the table. The *H. armigera* CYP9A12 enzyme of 0.5 mL, 1.5 mL, 2.5 mL and 3.5 mL were added which represented to 1%, 3%, 5% and 7% of CSM weight, respectively. The *H. armigera* CYP9A12 enzyme activities were 115 U/mg, 345 U/mg, and 575 U/mg, respectively. The concentration of the crude enzyme protein was 1.05 mg/mL and 230 U/mg, respectively. The concentration of the crude enzyme protein was 1.05 mg/mL and 230 U/mg, respectively (Bradford 1976). The enzymatic hydrolysis of the CSM was sampled after the reaction and dried in a vacuum freezer. Free and total gossypol content was determined by HPLC after crushing, sieving through 60-mesh, and supersonic extraction with acetone and butanone. The same treatment was repeated in three replicates.

**Table 1:** The level table of single test factor

Variable	A Initial temperature °C	B Enzymolysis time h	C Enzyme amount mL	D Substrate moisture %
1	25	1	0.5	35
2	30	2	1.5	40
3	35	6	2.5	45
4	40	12	3.5	50

#### Box–Behnken experimental design

The Box–Behnken experimental design is based on the mathematical model:

$$ Y = \beta_{0} + \sum {\beta_{i} x_{i} } + \sum {x_{i} x_{j} } + \sum {\beta_{ii} x_{i}^{2} } $$

*Y* is the response value (gossypol content); *β*_0_ is a constant term, *β*_*i*_ and *β*_*ii*_ are regression coefficients, and *x*_*i*_ and *x*_*j*_ are coded variables (temperature, time, enzyme addition and substrate moisture) (Ferreira et al. 2007). The contents of free gossypol in the detoxified CSM was used as the evaluation index in the Box–Behnken design. Each factor in the Box–Behnken design was coded as three levels with − 1, 0, and + 1 (Table 2). The quadratic regression fitting was conducted to get the quadratic equation with interactive terms and square terms using the corresponding code. The primary effects and interaction effects of each factor were analyzed. Finally, the optimal value was obtained within a certain level. One-way ANOVA analysis of multiple variables were carried out with the SPSS 17.0.

**Table 2:** Box–Behnken experimental factors and coding levels

Code	Variable	The coding level		
		− 1	0	1
A	Initial temperature °C	33	35	37
B	Enzymolysis time h	1.5	2	2.5
C	Enzyme additive amount mL	2.25	2.5	2.75
D	Substrate moisture %	38	40	42

## Results

### Detoxification effect of the *H. armigera* CYP9A12 enzyme in enzyme solution

The *H. armigera* CYP9A12 was obtained as described in our previous study (Chen et al. 2019). To validate the detoxification effect of *H. armigera* CYP9A12 in the enzyme reaction solution, the free and total gossypol contents were determined in the control group (without enzyme), endogenous group, and *H. armigera* CYP9A12 enzyme group, respectively. The results are shown in Table 3. The total gossypol was significantly decreased by 22.5% and 14.8% in comparison with the control group, respectively. The free gossypol content in solution was decreased by 2.5% and 2.6% after the addition of *H. armigera* CYP9A12 enzyme and endogenous enzyme, respectively.

**Table 3:** Effect of different treatments on gossypol content in cottonseed meal

Treatment	Enzyme reaction solution			
	Total gossypol (TG)	Detoxification rate (%)	Free gossypol (FG)	Detoxification rate (%)
Control group	34.25^a^ ± 3.35	–	27.53 ± 2.80	–
Endogenous group	29.16^b^ ± 2.37	14.8	26.81 ± 1.55	2.6
*H. armigera* CYP9A12	26.53^b^ ± 1.91	22.5	20.59 ± 2.54	2.5

	Enzymatic hydrolysis of cottonseed meal			
	Total gossypol (TG)	Detoxification rate (%)	Free gossypol (FG)	Detoxification rate (%)
Control group	147.21^a^ ± 0.88	–	110.23^a^ ± 1.13	–
Endogenous group	125.71^c^ ± 12.6	14.6	99.92^a^ ± 6.7	9.3
*H. armigera* CYP9A12	69.61^b^ ± 3.08	52.7	33.39^b^ ± 1.53	69.7
*Candida tropicalis*	53^b^.28 ± 3.65	63.8	42.32^c^ ± 5.52	61.6

Because gossypol is unstable and is easily oxidized, the P450 enzyme co-factor NADPH-Na_4_ was added to initiate the reaction and stabilize the gossypol. The ability of the *H. armigera* CYP9A12 enzyme to degrade gossypol was defined as the amount of one micromole of gossypol degraded per minute catalyzed by 1 mg of enzyme at 30 °C (pH 6). A unit of enzyme activity was expressed as U/mg. According to the definition of this enzyme activity, the concentration of *H. armigera* CYP9A12 enzyme protein in the supernatant of cell fragmentation induced by methanol for 72 h was 1.05 mg/mL, and the enzyme activity was 230 U/mg.

### Detoxification effect of *H. armigera* CYP9A12 enzyme on CSM

To validate the detoxification effect of *H. armigera* CYP9A12 enzyme in CSM, the free and total gossypol contents were determined in the control group, endogenous group, *H. armigera* CYP9A12 enzyme, and *C. tropicalis* group. The results are shown in Table 3. The free gossypol content in the CSM decreased by 52.7% and 63.8% after the addition of *H. armigera* CYP9A12 enzyme and *C. tropicalis* yeast (*p *< 0.05), respectively. Total gossypol significantly decreased by 69.7% and 61.6% for *H. armigera* CYP9A12 and *C. tropicalis*, respectively, in comparison to the control group (*p *< 0.05).

### Single factor test results of CSM enzymatic hydrolysis and detoxification by the *H. armigera* CYP9A12 enzyme

To optimize conditions of the *H. armigera* CYP9A12 enzyme on CSM, four different factors (initial temperature, enzymatic hydrolysis time, enzyme content, and substrate moisture) were planned in accordance with the abscissa, the ordinate free gossypol content for result analysis.

**Fig. 1:**
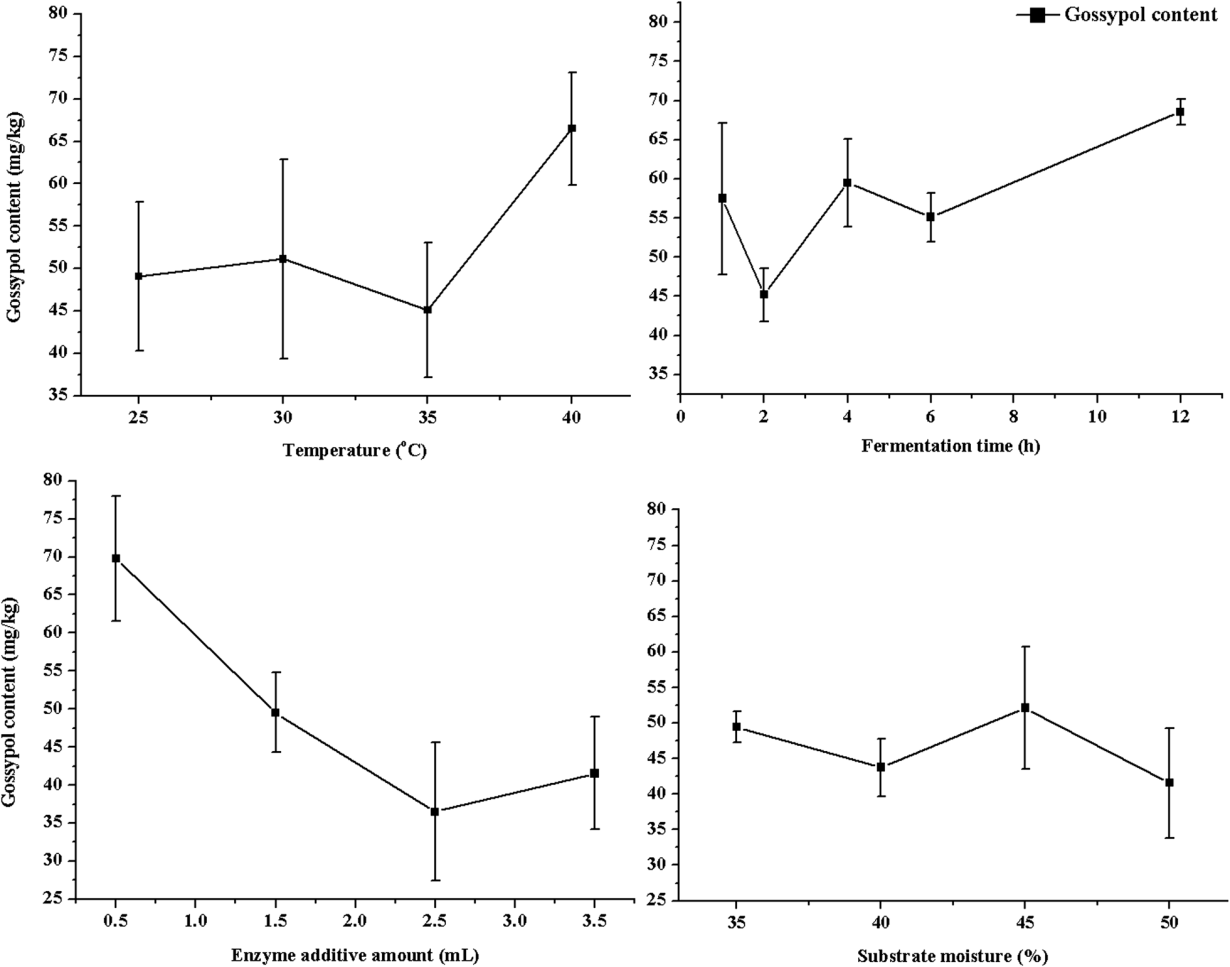
The single-factor test of the effect on gossypol of cottonseed meal by the *H. armigera* CYP9A12. The four different factors include initial temperature, enzymatic hydrolysis time, enzyme content, and substrate moisture. In accordance with the abscissa, the ordinate free gossypol content for concentration curve for result analysis. The optimal condition of the effect of *H. armigera* CYP9A12 enzyme on CSM gossypol degradation was achieved with 2.5 mL of enzyme. The enzymatic hydrolysis lasted 2 h at 35 °C, and the substrate moisture was 40%

The highest gossypol concentration in the enzymatic hydrolysis time was at 40 °C, and the lowest was observed at 35 °C. The hydrolysis time lasted 12 h, and the gossypol concentration was the highest, while the lowest was observed when the reaction time was 2 h. The highest and lowest contents of free gossypol were observed when the amount of enzyme was 0.5 and 2.5 mL, respectively. When the substrate moisture was 45%, the gossypol content was the highest. When the substrate moisture was 50%, gossypol concentration decreased to the lowest amount. In a single factor analysis test, the optimal condition of the effect of *H. armigera* CYP9A12 enzyme on CSM gossypol degradation was achieved with 2.5 mL of enzyme. The enzymatic hydrolysis lasted 2 h at 35 °C, and the substrate moisture was 40% (Fig. 1) in accordance with the abscissa, the ordinate free gossypol content for concentration curve for result analysis.

### Box–Behnken test results of CSM enzymatic hydrolysis and detoxification of *H. armigera* CYP9A12 enzyme

#### Model establishment and significance test analysis

Various factors do not exist independently in the process of enzymatic hydrolysis because they interact with each other. The response surface method was used to optimize the *H. armigera* CYP9A12 enzyme application conditions. The Box–Behnken test was designed using Design Expert software to quantify the effectiveness of key factors (initial temperature, enzymatic hydrolysis time, enzyme content, and substrate moisture) and the interactions among them. The free gossypol content was chosen as the response value *Y* for the purpose of this test. The independent variables, codes, and levels are shown in Table 2. The results obtained from 29 test points are shown in Table 4.

**Table 4:** Box–Behnken design and gossypol content

Treatment	Factor				Result
	A	B	C	D	Y mg/kg Gossypol content mg/kg
1	33	1.5	2.5	40	65.59
2	37	1.5	2.5	40	58.5
3	33	2.5	2.5	40	46.07
4	37	2.5	2.5	40	45.16
5	35	2	2.25	38	63.21
6	35	2	2.75	38	47.61
7	35	2	2.25	42	67.95
8	35	2	2.75	42	58.11
9	33	2	2.5	38	61.35
10	37	2	2.5	38	50.14
11	33	2	2.5	42	48.12
12	37	2	2.5	42	48.32
13	35	1.5	2.25	40	45.4
14	35	2.5	2.25	40	46.88
15	35	1.5	2.75	40	51.48
16	35	2.5	2.75	40	45.33
17	33	2	2.25	40	56.18
18	37	2	2.25	40	44.56
19	33	2	2.75	40	62.08
20	37	2	2.75	40	53.37
21	35	1.5	2.5	38	73.18
22	35	2.5	2.5	38	42.56
23	35	1.5	2.5	42	48.11
24	35	2.5	2.5	42	51.48
25	35	2	2.5	40	44.11
26	35	2	2.5	40	39.22
27	35	2	2.5	40	36.94
28	35	2	2.5	40	43.87
29	35	2	2.5	40	36.54

This table is the experimental results of Box–Behnken design and corresponding schemes. The gossypol content is selected as the response value Y. The independent variables A, B, C and D was corresponding to initial temperature, enzymatic hydrolysis time, enzyme content, and substrate moisture are shown in the table

Twenty-nine test points were divided into two categories. One category was a factorial point of 24 points corresponding to the independent variable values in A, B, C, and D constituting a three-dimensional vertex. The others were zero points in the center of the region, which had been repeated five times to estimate the test error. The results have shown the gossypol content of the CSM under the Box–Behnken experimental design (Table 4). The minimum and maximum gossypol contents were 36.54 and 73.18 mg/mL, respectively. The overall average value was 50.67 mg/mL; the overall standard deviation (SD) was 7.27, and the coefficient of variation (CV) was 7.35% meeting the requirements of the Box–Behnken analysis. The results were analyzed using multivariable regression fitting to obtain the quadratic polynomial regression equation of *Y* pairs of coded independent variables A, B, C and D.

$$ \begin{aligned} Y = & \, 3 7. 7 4- 3.0 3 {\text{A}} - 5. 40{\text{B}} - 0. 2 7 {\text{C}} - 0. 8 3 {\text{D}} \\ &+ 1. 5 4 {\text{AB}} + 0. 7 3 {\text{AC}} + 2. 10{\text{AD}} - 1. 9 1 {\text{BC}} + 8. 50{\text{BD}} \\ & + 2. 1 9 {\text{CD}} + 7. 3 2 {\text{A}}^{ 2} + 5. 2 4 {\text{B}}^{ 2} + 8. 4 1 {\text{C}}^{ 2} + 10. 2 9 {\text{D}}^{ 2} \\ \end{aligned} $$

The results showed that the experimental data in Table 4 were statistically significant (*p* = 0.0411, R^2^ = 0.7238), and the significant factor was the enzymatic hydrolytic time (B, *p* = 0.0221).

### Response surface analysis of gossypol on the effect of the *H. armigera* CYP9A12 enzyme on CSM

To obtain the range of responses for the four factors studied, two of the variables could be fixed at the central value, and the effects of the other two variables on gossypol content in CSM were analyzed and evaluated based on the response surface diagram and contour plot of the multivariate quadratic equation. The shape of the contours could reflect the intensity of the interaction effect. A circle indicates that the interaction between the two factors is not significant, but an ellipse indicates the interaction is significant.

**Fig. 2:**
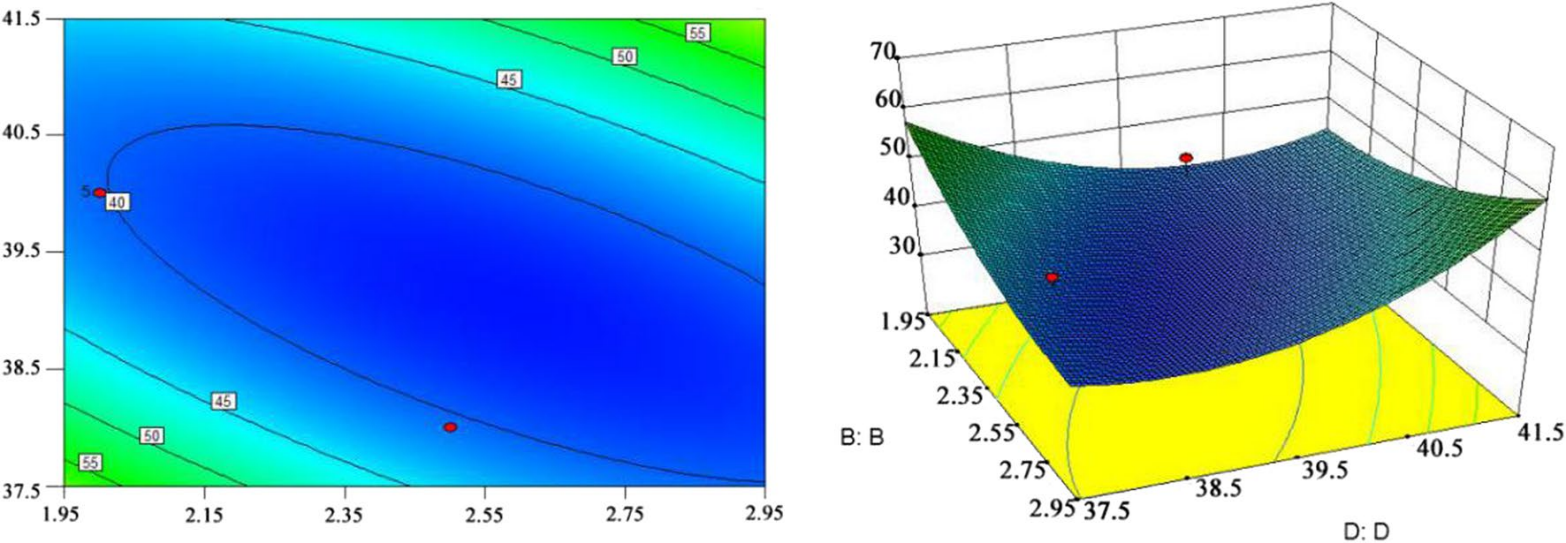
Contour plots and response surface diagram of the effect of reaction time (B) and cottonseed meal moisture (D) was analyzed on gossypol contents. The shape of contours could reflect the intensity of the interaction effect. The ellipse indicates that the interaction between the effect of reaction time (B) and cottonseed meal moisture (D) on gossypol content is significant

Results of the response surface graph and contour plot showed that factor B (enzymatic hydrolysis time) had a meaningful effect on gossypol content (*p* = 0.02), and the interaction between factor B and D was also important (*p* = 0.034) (Fig. 2). The interaction between the other factors was not significant difference. The predicted minimum gossypol content was 36.73 mg/kg under the following conditions: initial temperature 35.44 °C, enzymatic hydrolysis time 2.5 h, enzyme addition 2.55 mL and substrate moisture 39.23%. The predicted value was higher than the actual minimum value in the Box–Behnken experiment.

To verify the accuracy of the model, three experiments were conducted using the optimal conditions. The minimum gossypol content was 42.34 mg/kg, 40.72 mg/kg, and 39.68 mg/kg, with an average of 40.91 mg/kg. The results showed that the model could predict the optimal conditions for the *H. armigera* CYP9A12 enzymatic detoxification. After the optimization of the single factor and the Box–Behnken experimental design, the free gossypol content of the CSM decreased 88.4% from 352.94 to 40.91 mg/kg.

## Discussion

Gossypol is a natural phenolic compound that is derived from cotton plants. The toxicity of gossypol results from two active aldehyde groups which are toxic to most organisms (Nomeir and Abou-Donia 1985; Eisele 1986; Brocas et al. 1997; Chenoweth et al. 2000). The *H. armigera CYP6* and *CYP9* family genes *CYP321A1*, *CYP9A12*, *CYP9A14*, *CYP6AE11*, *CYP6B6* and *CYP6B7* are gossypol-inducible, which probably explains gossypol resistance of the *H. armigera* larvae (Yang et al. 1999; Celorio-Mancera et al. 2011; Mao et al. 2007, 2011; Tao et al. 2012; Zhou et al. 2010, Tian et al. 2017) or *Helicoverpa zea* larve (Stipanovic et al. 2006). Usually, the effects of the insect P450 enzymes on xenobiotics after expression in *E. coli* (Andersen et al. 1994; Guzov et al. 1998; Hayashi et al. 2003; Kaewpa et al. 2007; Liu et al. 2012), yeast (Pompon et al. 1996; Dietrich et al. 2005; Mirzaei et al. 2010) or insect *Sf9* cells (Krempl et al. 2016a, b) are nonfunctional when isolated from insect tissue (Andersen et al. 1994).

The *H. armigera* CYP6AE14 was solely expressed in *Sf9* cells but had no gossypol metabolic activity (Krempl et al. 2016a). However, the CYP6AE14 microsomes (Krempl et al. 2016a) and the coexpression of the NADPH P450 reductase from houseflies with *H. armigera* CYP6AE14 (Tao et al. 2012) had epoxidation activity towards aldrin. Presumably assistance of NADPH-cytochrome P450 reductase (CPR) to donate an electron was required. *H. armigera* CYP9A12 and CYP9A14 enzymes obtained from yeast have the ability to detoxify xenobiotics (Yang et al. 2008), but there have been no further metabolic studies related to gossypol.

After *H. armigera* CYP9A12 or endogenous enzyme treatment, free and total gossypol was extracted with 70% aqueous acetone and butanone, respectively, and prepared for HPLC analysis. The NADPH-Na_4_ was added to initiate the P450s enzyme reaction (Yang et al. 2004). Total gossypol content was equal to bound gossypol plus the free gossypol. Bound gossypol would be formed by free gossypol covalently binding to proteins in the reaction. Therefore, the endogenous group was assigned to remove the error. Total gossypol content decreased significantly from 34.25 to 29.16 and 26.53 μg/mL in the endogenous group and the *H. armigera* CYP9A12 group, respectively. In addition, free gossypol concentration decreased significantly from 27.53 to 26.81 and 20.59 μg/mL in the endogenous group and the *H. armigera* CYP9A12 group, respectively (Table 3). This value refers to the free gossypol that was metabolized by the *H. armigera* CYP9A12 enzyme rather than bound to the proteins.

**Fig. 3:**
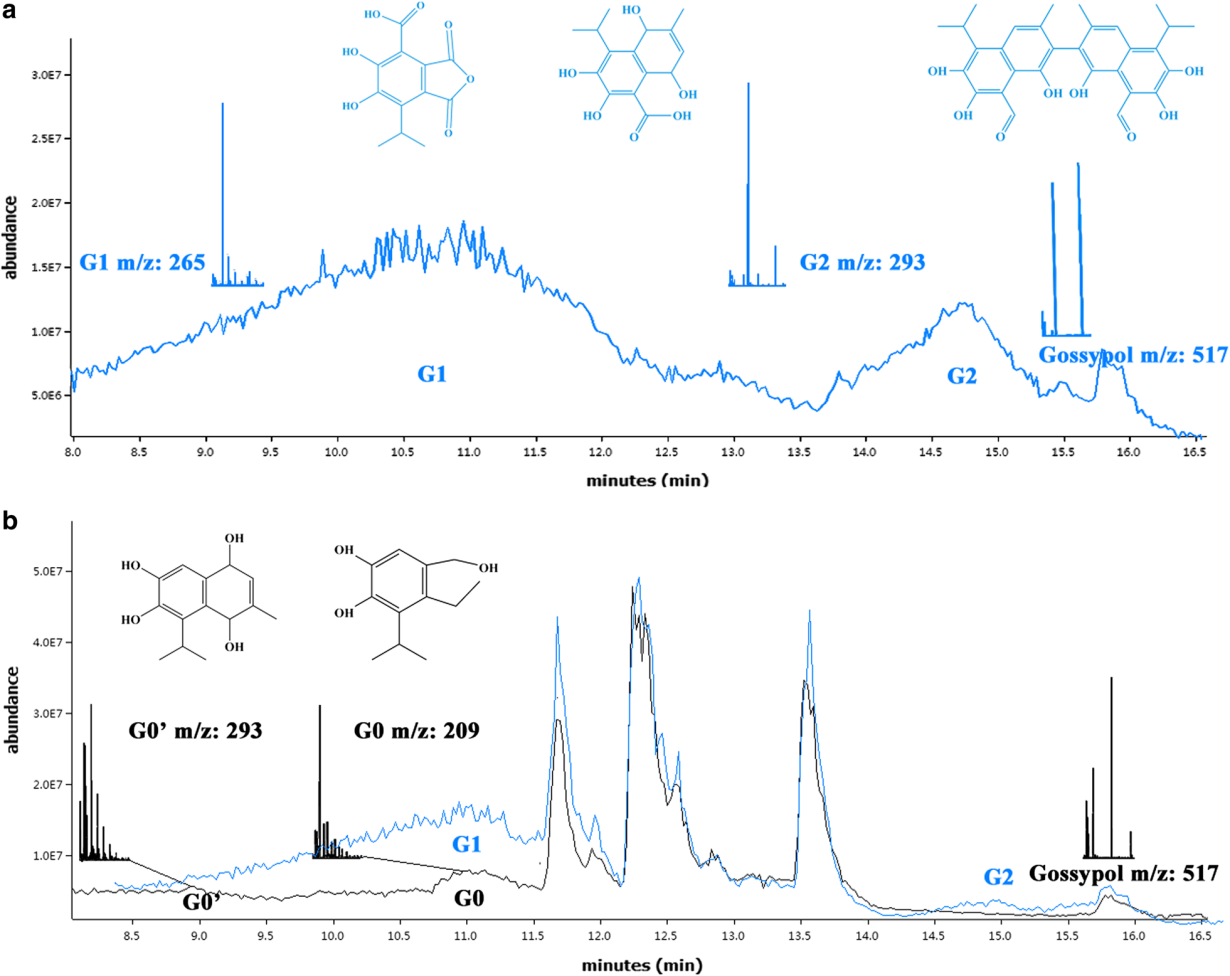
Extracted LC–MS ion chromatogram of the gossypol metabolites in negative ion mode and representative mass spectra of respective of chromatograms for quantification of gossypol and metabolites. **a** The control group without enzyme (blue) the free gossypol spontaneously degraded to compounds G1 (*m/z* 265) and G2 (*m/z* 293); **b** the recombinant of *H. armigera* CPR and CYP9A12 enzyme (black) was capable to degrade free gossypol by decarboxylation of G1 (*m/z* 265) and G2 (*m/z* 293) to compound G0 (*m/z* 209) and G0′ (*m/z* 249). The endogenous enzyme was as shown in blue (Chen et al. 2019)

Gossypol was characterized into gossypolone, gossypolonic acid, and demethylated gossic acid in pig livers (Abou-Donia and Dieckert 1975). The suggested oxidation pathway of gossypol (*m/z* 517) was first the formation of gossypolone (*m/z* 545) and subsequently gossypolonic acid (*m/z* 577), which was cleaved and oxidized to demethylated gossic acid (*m/z* 265) (Abou-donia and Dieckert 1974; Liu et al. 2014). Based on our previous study, the gossypol with an ion mass of 517.1910 would be spontaneously degraded to the gossypol metabolites, G1 and G2 with ion masses of 265.0411 and 293.1123. In addition, the two gossypol metabolites G1 and G2 would be degraded by *H. armigera* CYP9A12 enzyme into G0 and G0′ with ion masses of 209.0833 and 248.9578, respectively (Chen et al. 2019). Thus, the accumulation products G0′ and G0 were proposed to indirectly accelerate the metabolism of gossypol by the *H. armigera* recombinant CYP9A12 enzyme (Fig. 3).

Currently, the effective method of gossypol detoxification in CSM is microbial solid-state fermentation (Zhong and Wu 1989; Zhang et al. 2006a, b). However, the addition of corn flour, bran and other auxiliary materials and high-pressure sterilization during the drying process would result in the reduction of the free gossypol in solid-state processed CSM. The traditional solid-state fermentation of CSM has usually lasted for at least 2 days (Zhang et al. 2006b). It is difficult and costly to avoid the contamination of bacteria caused by excessive fermentation time. If the *H. armigera* recombinant CYP9A12 enzyme could be used in CSM, it could rapidly degrade gossypol content and greatly save time.

To validate the detoxification effect of the *H. armigera* CYP9A12 enzyme in CSM, free and total gossypol contents were determined in the control group, the endogenous group, the *H. armigera* CYP9A12 enzyme, and the *C. tropicalis* group. Before the experiment, an ultraviolet lamp was used to sterilize the CSM instead of autoclaving it. In addition, the experimental CSM was dried by vacuum freeze drying to avoid thermal influence on the gossypol content in CSM. The decrease of free gossypol in the CSM was degraded by microbial fermentation instead of binding with proteins to form bound gossypol (Wei et al. 2011). The free and total gossypol content in the CSM significantly decreased after the *H. armigera* CYP9A12 reaction and *C. tropicalis* fermentation (Table 3).

The optimal conditions of the four factors (temperature, time, enzyme addition and substrate moisture) were determined using the single-factor (Fig. 1) and response surface methods, respectively. Because the thermal stability of gossypol was below 40 °C, the temperature range of the *H. armigera* CYP9A12 enzyme detoxification of CSM was selected from 25 to 40 °C. However, in this experiment, the gossypol content in the CSM after processing was the highest when the temperature was 40 °C. We hypothesized that if the CSM in the enzymatic hydrolysis test was not sterilized after autoclaving, the growth of residual bacteria might lead to the increase in free gossypol content. We also found that the free gossypol content in the CSM was higher when the enzymatic hydrolysis temperature was 40 °C or the enzymatic hydrolysis time had lasted 12 h. A temperature of 37 °C and growth incubation time of 12 h are optimal conditions for *Bacillus subtilis* and *E. coli* growth, respectively. The optimal degradation time of *H. armigera* CYP9A12 enzyme in vitro was 30 min. Considering the existence of free gossypol in the CSM, the *H. armigera* CYP9A12 enzyme required full contact with the gossypol to degrade it. Therefore, the enzymatic hydrolysis time was set to 1, 2, 4, 6, and 12 h, respectively. The optimal enzymatic hydrolysis time for *H. armigera* CYP9A12 enzyme in CSM was 2 h. The coenzyme NADPH-Na_4_ was added to ensure the *H. armigera* CYP9A12 enzyme functioned. The free gossypol concentration in the CSM did not change significantly when the *H. armigera* CYP9A12 enzyme was added between 0.5 mL and 2.5 mL. The optimal CYP9A12 enzyme addition was determined to be 2.5 mL. When the substrate moisture was 50%, the gossypol level was the lowest, which could effectively increase the contact area between the *H. armigera* CYP9A12 enzyme and the gossypol in the CSM. Low moisture content does not help to decrease the gossypol, and excessive moisture would lead to additional drying and cost.

The *p* values of the primary and quadratic terms were less than 0.05 in the Box–Behnken response surface analysis, of which primary, secondary and interactive terms of the model equation were highly significant. Additionally, the CV value of this experimental design was 7.35 which reflects the confidence in the model, and the model’s ability to accurately reflect real test data. Based on the response surface diagram and the contour plot of the multivariate quadratic equation, factor B (enzymatic hydrolysis time) had a significant effect on gossypol content (*p* = 0.02) and the interaction between factor B and D (substrate moisture) (*p* = 0.034) influenced gossypol content (Fig. 2). After the predictive model and validation tests, the gossypol content could have reached a minimum of 40.91 mg/kg in CSM when the initial temperature was 35 °C; the enzymatic hydrolysis time had lasted 2.5 h; the enzyme addition was 2.5 mL, and the substrate moisture was 39%.

At present the detoxification effect of recombinant CYP450s on cottonseed meal was still limited by the cost of the co-factor NADPH and the enzyme yield. If the enzyme could be further highly expressed by heterologous system and in industrial production which could greatly increase the rate of gossypol degradation and provide a new strategy for cottonseed meal detoxification.

The recombinant *H. armigera* CYP9A12 and its reductase were successfully expressed in the *P. pastoris* system. The CYP9A12 was able to accelerate metabolism of the gossypol intermediate metabolites. Treatment of CSM with *H. armigera* CYP9A12 enzyme significantly degraded free and total gossypol. After optimization of the single-test and response surface method, the free gossypol content could decrease to 40.91 mg/kg in the CSM when the initial temperature was 35 °C, the enzymatic hydrolysis time lasted 2.5 h, the enzyme addition was 2.5 mL, and the substrate moisture was 39%.

## Data Availability

All data generated or analyzed during this study are included in this published article.

## Abbreviations

G0′ to G2: gossypol metabolite 0′ to gossypol metabolite 2
CPR: NADPH-cytochrome P450-reductase
CYP9A12: cytochrome P450-monooxygenase CYP9A12
CSM: cottonseed meal
*H. armigera*: *Helicoverpa armigera*
*P. pastoris*: *Pichia pastoris*
GA: Gibson assembly
